# Amiloidose para Cardiologistas

**DOI:** 10.36660/abc.20210959

**Published:** 2022-02-14

**Authors:** Roberto Coury Pedrosa

**Affiliations:** 1 Departamento de Cardiologia Hospital Universitário Clementino Fraga Filho Universidade Federal do Rio de Janeiro Rio de Janeiro RJ Brasil Departamento de Cardiologia do Hospital Universitário Clementino Fraga Filho / Instituto do Coração Edson Saad / CEPARM. Centro Nacional de Referência em Amiloidose Brasileira – Universidade Federal do Rio de Janeiro,Rio de Janeiro, RJ – Brasil

**Keywords:** Cardiomiopatia Amiloidótica

Atualmente, sabemos que a amiloidose cardíaca (AC) é mais frequente do que tradicionalmente considerada e que é particularmente relevante em pacientes acima de 65 anos de idade com insuficiência cardíaca ou estenose aórtica.^1^ Hoje, dada a relevância da AC para cardiologistas, sua prevalência ainda é um problema e esforços devem ser feitos para acelerar o diagnóstico e maximizar as oportunidades de novos tratamentos modificadores da doença.^1 - 3^ No Brasil, estima-se que existam mais de 5.000 pacientes com a variante da amiloidose por transtirretina (ATTRv) com polineuropatia,^4^ onde o envolvimento cardíaco também desempenha um papel importante. Verificamos que 26% dos pacientes com V30M ATTRv-PN do nosso centro de referência registrados no THAOS eram casos de início tardio (LO).^5^ Nesses pacientes, encontramos hipertrofia do septo interventricular em quase 70% e ECG anormal em quase 90%. É de interesse que 78% dos pacientes com LO-V30M e cardiomiopatia não apresentaram sintomas de insuficiência cardíaca.^5^

No nosso país, não há políticas de saúde públicas ou privadas destinadas especificamente ao monitoramento e acompanhamento dos pacientes com AC. Da mesma forma, pouco se sabe sobre as repercussões dessa doença nas taxas de mortalidade.^4 - 8^ Muitas barreiras estruturais são difíceis de superar, tais como, a falta de implementação de programas de rastreamento e de testes diagnósticos validados, principalmente em áreas rurais. Não há entidade que monitore esses processos e os casos positivos podem não receber confirmação ou acesso ao tratamento. O sequenciamento genético do gene TTR é uma ferramenta nova, mas ainda não está disponível em laboratórios gerais, estando restrito à pesquisa clínica em hospitais ou universidades. Acreditamos que muitos pacientes com AC morrem dessa doença devido à falta de assistência médica adequada.^4 , 7^ Consideramos que seja urgente adotar medidas que possam melhorar esta situação.

As diretrizes clínicas das sociedades médicas têm sido atualizadas. Mais recentemente a Sociedade Brasileira de Cardiologia,^9^ a Sociedade Europeia de Cardiologia^10^ e a American Heart Association^11^ emitiram posicionamentos sobre AC, documentos com informações atualizadas incluindo diferentes aspectos da doença, algumas barreiras identificadoras, e soluções potenciais para cada etapa da doença. Infelizmente, essas diretrizes ainda não foram totalmente disseminadas ou implementadas.

Um dos aspectos desafiadores do manejo da AC é a identificação de pacientes que apresentam essa condição. É necessário garantir que todos os médicos que possam encontrar esses pacientes saibam o que procurar, não apenas no histórico dos pacientes, mas também em ecocardiogramas, estudos de ressonância magnética e cintilografia com pirofosfato. Na presente edição, Fernandes et al.^12^ apresentaram um estudo brasileiro que contribui para o nosso entendimento sobre uma importante característica da amiloidose sistêmica, ou seja, a falta de dados na população brasileira a respeito da prevalência e gravidade do acometimento cardíaco. Para responder a esta questão, os autores realizaram um estudo retrospectivo de uma amostra de conveniência de pacientes acompanhados em um centro de referência em cardiologia de um hospital terciário brasileiro, com diagnóstico confirmado de amiloidose sistêmica com cardiomiopatia, e indivíduos de outras unidades de saúde e diferentes especialidades (neurologia, hematologia, nefrologia e gastroenterologia) para avaliação do envolvimento cardíaco (cardiomiopatia amiloidótica) da doença já confirmada em outros órgãos e sistemas. O objetivo foi descrever o perfil clínico, laboratorial, eletrocardiográfico e de imagem dos pacientes com AC.

Foram avaliados 105 pacientes (idade mediana de 66 anos); 83 tinham amiloidose transtirretina (ATTR) e 22 amiloidose de cadeia leve (AL). Em relação aos casos de ATTR, 68,7% eram da forma hereditária (ATTRv) e 31,3% eram do tipo selvagem (ATTRw). As mutações mais prevalentes foram V142Ile (45,6%) e V30M (40,3%). O tempo desde o início dos sintomas até o diagnóstico foi de 0,54 e 2,15 anos, nas formas AL e ATTR, respectivamente (p < 0,001). O envolvimento cardíaco foi observado em 77,9% dos pacientes com ATTR e em 90,9% daqueles com AL.

Na interpretação desses resultados, é necessário considerar o desenho do estudo (amostra de conveniência), com coleta retrospectiva de dados, que representa uma amostra parcial da população de pacientes atendidos neste centro único para AC, sem cálculo do tamanho da amostra ou pareamento para variáveis importantes como idade e sexo. A amostra é constituída por uma subpopulação submetida a exames de imagem cardíaca; portanto, não representa o cenário real da população com AC, mas sim um subgrupo selecionado com o melhor prognóstico para o qual o médico assistente indicou a realização de exames de imagem cardíaca. Os autores reconhecem essas limitações.

É importante enfatizar que, no processo dinâmico de envolvimento cardíaco em pacientes com AC suspeita e/ou confirmada, é de extrema importância identificar, com poucos recursos, pacientes com AC com maior risco de ocorrência de óbito ou eventos cardíacos recorrentes. Isso se traduz em monitoramento da frequência cardíaca e do remodelamento ventricular.

Mais importante, as complicações cardiovasculares da AC ao longo do tempo são inevitáveis, e nós, como cardiologistas, devemos considerar que todos os pacientes com CA podem se beneficiar de diretrizes direcionadas para disfunção sistólica do VE. A responsabilidade dos profissionais de saúde, das sociedades médicas, das organizações de pacientes e dos legisladores é trabalhar junto a longo prazo para mudar o conceito de que a AC seja uma doença intratável.
