# Supplementary material for: Amiloidose para Cardiologistas
Source: Arq Bras Cardiol. 2022 Feb 14;118(2):433–4. [Article in Portuguese] doi: 10.36660/abc.20210959 (PMC8856688; doi:10.36660/abc.20210959)
Supplement: Translation

# Translation: Amyloidosis for Cardiologists

**Authors:** Roberto Coury Pedrosa

**Affiliations:** 1 Brazil Departamento de Cardiologia do Hospital Universitário Clementino Fraga Filho / Instituto do Coração Edson Saad / CEPARM. Centro Nacional de Referência em Amiloidose Brasileira – Universidade Federal do Rio de Janeiro, Rio de Janeiro, RJ – Brazil

**Keywords:** Amyloidosis Cardiomyopathy

We now know that cardiac amyloidosis (CA) is more frequent than traditionally considered and that it is particularly relevant in patients over 65 years with heart failure or aortic stenosis.^1^ Nowadays, given the relevance of CA for cardiologists, its prevalence is still a problem, and efforts must be made to speed up diagnosis and maximize opportunities for new disease-modifying treatments.^1 - 3^ In Brazil, it is estimated that there are more than 5,000 patients with the transthyretin amyloidosis variant (ATTRv) with polyneuropathy,^4^ where cardiac involvement also plays an important role. We found that 26% of patients with V30M ATTRv-PN from our referral center registered in THAOS are late-onset cases (LO).^5^ In these patients, we found interventricular septum hypertrophy in almost 70% and abnormal ECG in almost 90%. Interestingly, 78% of the patients with LO-V30M and cardiomyopathy did not have symptoms of heart failure.^5^

In our country, there are neither public nor private health policies specifically designed to monitor and follow-up patients with CA. Similarly, very little is known about the repercussions of this disease on mortality rates.^4 - 8^ Many structural barriers are difficult to overcome, such as lack of implementation of screening programs and lack of validated diagnostic tests, particularly in rural areas. There is no entity that monitors these processes, and positive cases may not receive confirmation or access to treatment. The genetic sequencing of the TTR gene is a new tool, but it is not yet available in general laboratories; it is restricted to clinical research in hospitals or universities. We believe that many patients with CA die from this disease due to lack of appropriate and adequate medical care.^4 , 7^ We consider it urgent to adopt measures that can improve this situation.

Clinical guidelines of the medical societies have been updated. Recently, the Brazilian Society of Cardiology,^9^ the European Society of Cardiology,^10^ and the American Heart Association^11^ issued position statements regarding CA, documents with updated information including different aspects of the disease, some identifying barriers, and potential solutions for every step of the disease. Unfortunately, these guidelines have not yet been fully disseminated or implemented.

One of the challenging aspects of managing CA is the identification of patients who have this condition. It is necessary to ensure that all clinicians who may encounter these patients know what to look for, not only in the patients’ history but also in echocardiograms, magnetic resonance imaging studies, and pyrophosphate scans. In this edition, Fernandes et al.^12^ presented a Brazilian study that contributes to our understanding of an important feature in systemic amyloidosis, i.e., lack of data in the Brazilian population regarding the prevalence and severity of cardiac involvement. To answer this question, the authors performed a retrospective study of a convenience sample of patients monitored at a reference cardiology center in a tertiary hospital in Brazil, with confirmed diagnosis of systemic amyloidosis with cardiomyopathy, and individuals from other health units and different specialties (neurology, hematology, nephrology, and gastroenterology) for evaluation of cardiac involvement (amyloidosis cardiomyopathy) of the disease already confirmed in other organs and systems. The objective was to describe the clinical, laboratory, electrocardiographic, and imaging profile of patients with CA.

A total of 105 patients were evaluated (median age of 66 years); 83 had transthyretin (ATTR) amyloidosis, and 22 had light chain (AL) amyloidosis. With respect to ATTR cases, 68.7% were the hereditary form (ATTRv), and 31.3% were wild type (ATTRw). The most prevalent mutations were V142Ile (45.6%) and V30M (40.3%). Time from onset of symptoms to diagnosis was 0.54 and 2.15 years, in the AL and ATTR forms, respectively (p < 0.001). Cardiac involvement was observed in 77.9% of patients with ATTR and in 90.9% of those with AL.

When interpreting these results, it is necessary to consider the design of the study (convenience sample), with retrospective data collection, which represents a partial sample of the population of patients assisted in this single center for CA, without sample size calculation or matching for important variables such as age and sex. It comprises a subpopulation who underwent cardiac imaging exams; therefore, it does not represent the real scenario of the population with CA, but rather a subgroup selected with the best prognosis for which the attending physician indicated the performing cardiac imaging exams. The authors acknowledge these limitations.

It is important to emphasize that, in the dynamic process of cardiac involvement in patients with suspected and/or confirmed CA, it is extremely important to identify, with few resources, patients with CA at higher risk for the occurrence of death or recurrent cardiac events. These translate into heart rate monitoring and ventricular remodeling.

More importantly, cardiovascular complications of CA over time are unavoidable, and we, as cardiologists, must consider that all patients with CA benefit from guideline-directed medical therapy for LV systolic dysfunction. The responsibility of healthcare professionals, medical societies, patient organizations, and policymakers is to work together over the long term to change the concept that CA is an intractable disease.
